# Specific protein carbonylation in human breast cancer tissue compared to adjacent healthy epithelial tissue

**DOI:** 10.1371/journal.pone.0194164

**Published:** 2018-03-29

**Authors:** Baikuntha Aryal, V. Ashutosh Rao

**Affiliations:** Laboratory of Applied Biochemistry, Division of Biotechnology Review and Research III, Office of Biotechnology Products, Center for Drug Evaluation and Research, U.S. Food and Drug Administration, Silver Spring, Maryland, United States of America; University of South Alabama Mitchell Cancer Institute, UNITED STATES

## Abstract

Protein carbonylation is an irreversible post-translational modification induced by severe oxidative stress. Reactive oxygen species (ROS) are constantly produced in cells and play important roles in both cancer progression and cancer suppression. ROS levels can be higher in tumor compared to surrounding healthy tissue but ROS-induced specific protein carbonylation and its unique role in cancer progression or suppression is poorly understood. In this study, we utilized previously validated ELISA and western blot methods to analyze the total and specific protein carbonylation in flash-frozen human breast cancer and matched adjacent healthy tissue to compare relative total, and specific protein carbonylation. Mass spectrometry, two-color western, and immunoprecipitation methods were used to identify and confirm the specifically carbonylated proteins in breast tumor tissue. Superoxide dismutase (SOD) activity was measured as an indicator of antioxidant activity, and LC3-II protein level was analyzed for autophagy by western blot. Findings were further confirmed using the immortalized MDA-MB-231 and MDA-MB-468 breast cancer and MCF-12A noncancerous human epithelial breast cell lines. Our results indicate that tumor tissue has greater total protein carbonylation, lower SOD1 and SOD2 protein levels, lower total SOD activity, and higher LC3-II levels compared to adjacent healthy tissue. We identified and confirmed three specific proteins of interest; filamin A, heat shock protein 90β (HSP90β), and bifunctional glutamate/proline-tRNA ligase (EPRS), that were selectively carbonylated in tumor tissue compared to matched adjacent healthy tissue. Correspondingly, compared to noncancerous MCF-12A epithelial cells, MDA-MB-231 cancer cells exhibited an increase in filamin A and EPRS protein carbonylation, decreased total SOD activity, and increased autophagy, but not increased HSP90β protein carbonylation. Identification of selectively carbonylated proteins and defining their roles in cancer progression may promote the development of targeted therapeutic approaches toward mitigating oxidative damage of these proteins.

## Introduction

A multitude of mechanisms and factors influence the origin and development of breast cancer. Oxidative stress is one of the factors associated with the initiation and progression of this disease [1]. Reactive oxygen species (ROS) are normal byproducts of metabolism due to incomplete one-electron reduction of oxygen in the mitochondrial electron transport chain and play a vital role in cell signaling pathways. Cellular antioxidant systems consistently neutralize excess ROS to maintain optimal levels for cellular function while an imbalance between ROS production and its neutralization leads to oxidative stress. Low antioxidant enzyme activity including superoxide dismutase (SOD) and high levels of oxidative stress have been reported in various cancer cell lines and tissues compared to healthy counterparts [2–5]. Under constant environmental stress, overproduction of ROS may alter cellular structure and function by somatic mutation leading to neoplastic transformation [6]. In addition to inducing genetic instability, excessive ROS can activate several proliferative signaling pathways for tumor development [7]. In cancer cells, ROS and antioxidant levels are tightly regulated to promote tumorigenesis while avoiding excessive ROS-induced detrimental effects [8]. Thus, proposed therapeutic strategies for chemotherapy often involve either application of antioxidants to deplete ROS-induced survival signaling pathways or ROS generating agents to induce irreparable damage and tumor cell apoptosis depending upon the types and stages of cancer, level of endogenous ROS, and abundance of ROS-induced survival pathways [9].

Excessive free radicals can cause oxidative damage to proteins, nucleic acids and lipids. Several types of oxidative modifications in proteins, caused by free radicals, have been reported. Investigations into oxidative stress-induced protein modification have benefited from recent advances in analytical methods [10, 11]. Distinct from methionine or cysteine modification, protein carbonylation is an irreversible modification commonly occurring at the side chain of proline, arginine, lysine, and threonine residues. Carbonylation is induced by all types of ROS *in vivo* and tags proteins for proteasomal degradation, making it a clinically relevant modification [10]. Oxidative stress-induced carbonyl modification of proteins has several structural and functional consequences including loss of protein function, abnormal protein clearance, alteration in cellular redox balance, interference with cell cycle, and cancer progression [12, 13]. In agreement with elevated levels of oxidative stress in cancerous cells, higher total protein carbonylation has been reported in several types of cancer [14, 15]. Using mass spectrometry analysis, some specifically carbonylated low molecular weight proteins (<80 kDa) have been identified in cholangiocarcinoma [13]. However, a systematic study of protein carbonylation comparing differences between breast cancer and healthy tissue has not been reported. Identification of specific proteins that are carbonylated and proteasomally degraded in tumor, but not in healthy tissue, will further our understanding of the etiology of breast cancer and potentially identify targets for preventing or treating the disease. Western blot analysis indicates that proteins with a molecular mass greater than 50 kDa are more susceptible to oxidation via carbonylation compared to low molecular weight proteins in tumor tissue, but previous studies have identified only low molecular weight carbonylated proteins (<80 kDa) in cholangiocarcinoma tissue [13]. In this study, we employed a combination of 1D gel electrophoresis, two–color western blot, mass spectrometry, and immunoprecipitation to analyze and identify specifically carbonylated high molecular weight proteins in human breast cancer tissues compared to matched adjacent healthy tissues.

## Materials and methods

### Preparation of tissue lysate

Four pairs of banked human flash-frozen breast tumor and adjacent healthy tissue were purchased from Capital Biosciences (Rockville, MD) and Cureline Inc. (San Francisco, CA). This study involved the use of existing de-identified specimens and did not require a Research Involving Human Subjects Committee (RIHSC) review and approval because it is exempt from the requirements of 45 CFR §46.101b(4). The study was conducted with adherence to the policies and procedures set forth by the RIHSC which is the Food and Drug Administration’s institutional review committee primarily concerned with the ethical and human protection aspect of the intramural research and protocols involving human subjects. All commercially-obtained samples were analyzed anonymously and previously collected by surgical procedure, had no record of chemo- or radiation therapy and histologically designated as ductal infiltrative cancer by the vendor.

Approximately 50 mg of tissue was excised from the frozen tumor and adjacent healthy tissue samples and washed with ice-cold PBS containing protease inhibitor cocktail (Roche Diagnostics Corporation, Inc., Indianapolis, IN) and phosphatase inhibitor (Sigma–Aldrich, St Louis, MO). The samples were then homogenized in radioimmunoprecipitation assay (RIPA) buffer (Thermo Scientific, Grand Island, NY) or whole cell lysis buffer (20 mM Tris-HCl pH 7.5, 150 mM NaCl, 1 mM EGTA, 1% (v/v) Triton X-100, 1 mM Na_3_VO_4_) containing protease inhibitor cocktail and phosphatase inhibitor. The tissue homogenates were incubated on ice for 20 minutes, and centrifuged at 12,000 x *g* for 20 minutes at 4°C. Supernatant was collected in a clean tube and protein concentration was determined using BCA protein assay kit (Thermo Scientific, Rockford, IL). Tissue lysate was aliquoted and stored at -80°C for biochemical analysis.

### Determination of total protein carbonylation by ELISA

Quantification of protein carbonylation was performed using a modified ELISA method as described in a previous publication by our group [11]. Briefly, 10 μL of 1 μg/μL of protein lysate from each sample was denatured with 10 μL of 10% (w/v) sodium dodecyl sulfate (SDS) and derivatized with 20 μL of 20 mM 2,4-dinitrophenylhydrazine (DNPH) solution prepared in 10% (v/v) trifluoroacetic acid (TFA). After incubation at room temperature for 10 minutes with vortexing every 2 minutes, the reaction was neutralized with 20 μL of 2 M Tris base. A 3 μL aliquot of DNP-derivatized sample was diluted with 0.25 mL of adsorption buffer (20 mM NaHCO_3_, 150 mM NaCl, 0.25% SDS (w/v), pH 8.5), and 100 μL of diluted sample was loaded on to a 96-well Maxisorp plate. The plate was covered with aluminum foil and incubated overnight at 4°C. After incubation, the sample wells were rinsed gently 6 times with PBST (1X PBS containing 0.05% Tween 20) and incubated with 200 μL of blocking buffer (1% BSA in adsorption buffer) for 1 hour at 37°C. The sample wells were then incubated with 100 μL of blocking buffer containing goat anti-DNP antibody for 1 hour at room temperature. Following incubation, the sample wells were rinsed 6 times with PBST, and incubated with horseradish peroxidase (HRP)-conjugated rabbit anti-goat IgG antibody for 1 hour at room temperature. After washing 6 times with PBST, sample wells were incubated with 100 μL of TMB substrate at room temperature for 2–3 minutes for color development. The reaction was stopped with 100 μL of 0.5 M H_2_SO_4_ and the absorbance was measured at 450 nm and 690 nm. After subtraction of background absorbance at 690 nm, the carbonyl content in each sample was determined using a standard curve of oxidized BSA standard.

### Determination of protein carbonylation by western blot

Protein carbonylation was determined by derivatization of protein carbonyls with DNPH using a procedure based on the previous publications [10, 16]. Approximately 10 μg of protein lysate for each sample was treated with 6% (w/v) SDS in a 15 μL volume. An equal volume of 20 mM DNPH in 10% (v/v) TFA was added and incubated at room temperature for 10 minutes. The reaction was neutralized with 15 μL of 2 M Tris in 30% (v/v) glycerol containing 7% (v/v) β-mercaptoethanol, and 15 μL of DNP-derivatized sample was loaded in each lane of two identical gels (4–12% bis-tris or 3–8% tris-acetate). One gel was used for Coomassie staining and the other for western blot analysis to determine specific protein carbonylation. Proteins from the gel were transferred to an immobilon-P PVDF membrane (Millipore, Billerica, MA). After blocking for 1 hour with blocking buffer (LI-COR, Lincoln, NE), the membrane was incubated with goat anti-DNP primary antibody (Bethyl Laboratories Inc., Montgomery, TX) followed by donkey anti-goat IRDye 800CW secondary antibody (LI-COR, Lincoln, NE). The DNP-derivatized carbonylated proteins were detected using the Odyssey infrared imaging system (LI-COR, Lincoln, NE).

Western blot analysis for LC3-II, SOD1, and SOD2 was performed using anti-LC3-II (Novus Biologicals, Littleton, CO), anti-SOD1 (Millipore, Billerica, MA), anti-SOD2 (Abcam, Cambridge, MA), and β-actin (Santa Cruz Biotechnology, Dallas, TX) antibodies.

### Protein identification

Protein bands in the silver stained gel corresponding to highly carbonylated proteins in the western blot were excised, and digested with sequencing grade modified trypsin (Promega Corporation, Madison, WI) for 16 hours at 37°C per manufacturer’s instructions. The trypsin-digested peptides were then subjected to liquid chromatography-tandem mass spectrometry (LC-MS/MS) analysis using Q-TOF LC/MS (Agilent Technologies, Santa Clara, CA). Peptide search and protein identification were performed using the Spectrum Mill MS Proteomic Workbench (Agilent Technologies, Santa Clara, CA). Trypsin-digested bovine serum albumin (BSA) was used as a quality control for MS analysis.

### Two-color western

Two-color western blot analysis was utilized to confirm proteins identified by mass spectrometry. After transfer, the PVDF membrane was incubated with two primary antibodies; a goat anti-DNP antibody and a rabbit or mouse antibody against the proteins of interest for 3 hours at room temperature. The membrane was washed three times with PBST and incubated with two secondary antibodies; a donkey anti-goat 800CW (green) for carbonyl detection and a donkey anti-rabbit or anti-mouse 680LT (red) for 1 hour at room temperature to detect carbonylated proteins of interest. DNP-derivatized carbonylated proteins (green) and specific proteins of interest (red) were detected using the Odyssey infrared imaging system (LI-COR, Lincoln, NE). The identity and degree of carbonylation of each protein of interest were confirmed with the overlapping green and red bands on the blot. A similar method was used to analyze the specific carbonylated proteins in the MDA-MB-231 breast cancer cell line and MCF-12A noncancerous healthy human epithelial breast cell line by two-color western.

### Immunoprecipitation

An immunoprecipitation assay was performed for all three proteins identified by LC-MS/MS and two-color western using antibodies against specific proteins and protein A/G PLUS agarose beads (Santa Cruz Biotechnology, Inc., Dallas, Texas). About 300 μg of protein lysate was incubated with antibody against the protein of interest for 3 hours followed by incubation with A/G PLUS agarose beads overnight at 4°C. The beads were washed four times with lysis buffer containing protease inhibitor and the protein of interest from each sample was extracted in 10 mM Tris-HCl pH 7.5 containing 6% SDS. The extracted proteins were derivatized with DNPH as previously described and resolved by SDS-PAGE. Two-color western blot analysis was performed using a goat anti-DNP antibody and an antibody against the protein of interest to determine the carbonyl content in each specific protein.

### Superoxide dismutase activity

Tissue homogenate from tumor and adjacent tissue was prepared in ice cold 20 mM HEPES at pH 7.2, containing 1 mM EGTA, 210 mM mannitol, and 70 mM sucrose. Protein concentration was determined by BCA assay using BSA as a protein standard. SOD activity was determined using a kit from Cayman Chemical (Ann Arbor, MI) according to the manufacturer’s instructions. This kit utilizes a tetrazolium salt to detect superoxide radicals generated by xanthine oxidase and hypoxanthine. In the presence of superoxide, the tetrazolium salt is converted to a water-soluble formazan dye that can be monitored by absorbance at 440–460 nm. SOD activity in MDA-MB-231 and MCF-12A cell lines was determined in an analogous way.

## Results

### Total and specific protein carbonylation in breast tumor tissue

**Fig 1:**
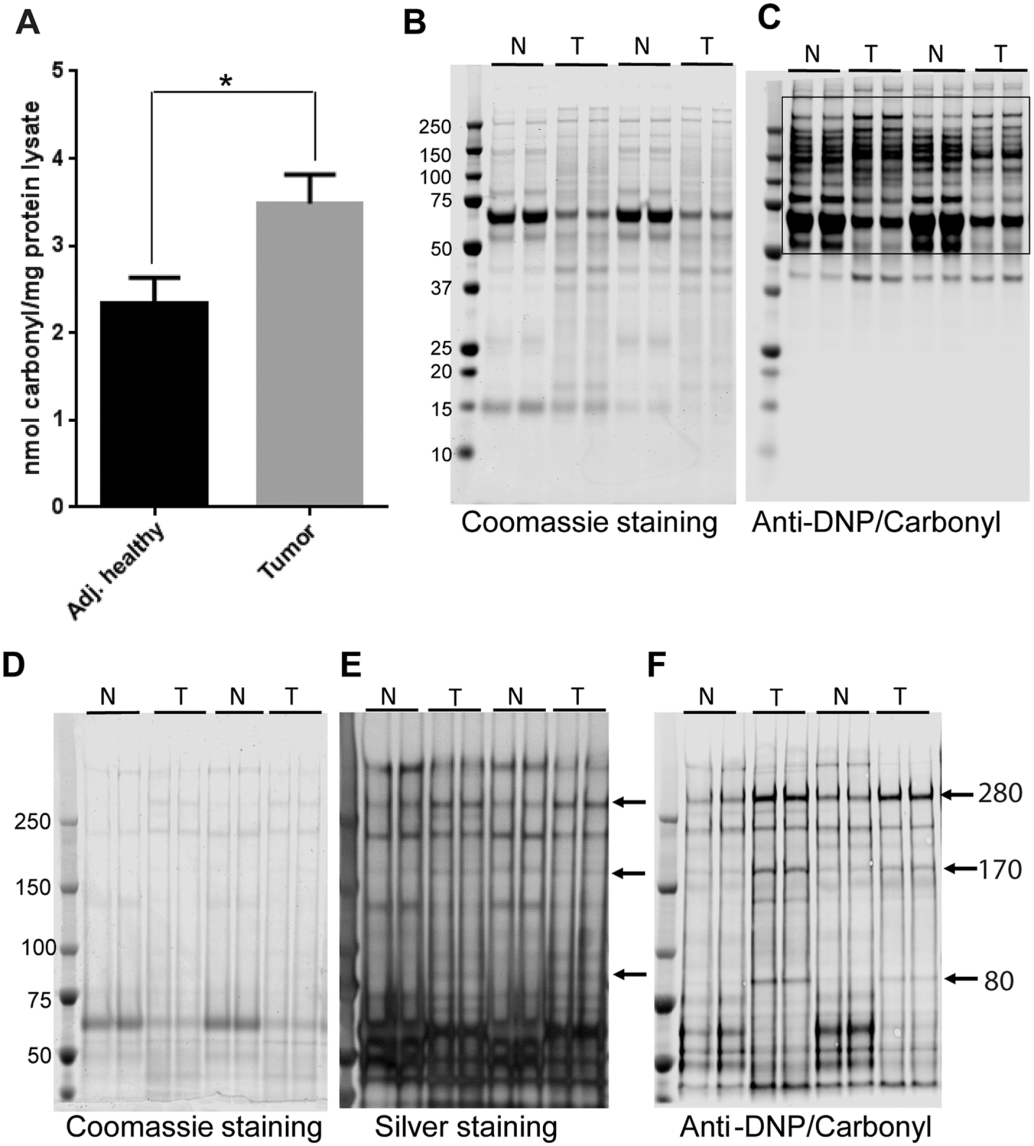
Total and specific protein carbonylation in human breast cancer and adjacent healthy tissue. (A) Quantification of total protein carbonylation in tumor and adjacent healthy tissue using ELISA method. (B) Representative Coomassie blue stained gel (4–12% bis-tris gel) and (C) western blot for carbonylated proteins using anti-DNP antibody. (D) Representative Coomassie blue stained gel (3–8% tris-acetate gel), (E) silver stained gel, and (F) western blot for carbonylated proteins. Arrows represent three specifically carbonylated proteins in human breast cancer tissue with expected molecular weights of 280 kDa, 170 kDa, and 80 kDa. Each sample was loaded in duplicate. N = adjacent healthy tissue, T = breast tumor tissue.

We first established the total level of protein carbonylation in tumor and adjacent healthy tissue using an ELISA method. Consistent with the majority of previously reported results [13–15, 17] we observed greater protein carbonylation in lysates from tumor tissue compared to their matched adjacent healthy counterparts (Fig 1A). To determine the comparative specific protein carbonylation profile in tumor and adjacent tissue, we used a 4–12% bis-tris gel to resolve proteins and performed western blot analysis using anti-DNP antibody. While there were some detectable carbonylated protein bands of low molecular weight proteins on the western blot (MW less than 50 kDa), we noticed an appreciable level of protein carbonylation in the higher molecular weight region of the western blot (Fig 1B and 1C). To better resolve and compare the higher molecular weight proteins we then used a 3–8% tris-acetate gel. Western blots showed three specific proteins that were distinctly carbonylated, to a greater extent, in tumor compared to adjacent healthy tissue (Fig 1D–1F). We analyzed four pairs of tissue lysate and all samples showed higher carbonylation of three specific proteins in tumor tissues compared to adjacent healthy tissue. Only a small amount of protein lysate (2.5 to 3.5 μg/lane) was required to achieve a high resolution western blot for the DNP-derivatized proteins, but protein bands were nearly undetectable in a Coomassie blue stained gel with such a low amount of protein loaded (Fig 1D). Therefore, we used silver staining to visualize protein bands in the gel (Fig 1E). The silver stained gel showed bands corresponding to the anti-DNP reactive carbonyl bands at 280 kDa, 170 kDa, and 80 kDa on the western blot (Fig 1E).

### Identification of carbonylated proteins by LC-MS/MS analysis, immunoprecipitation, and two–color western

**Fig 2:**
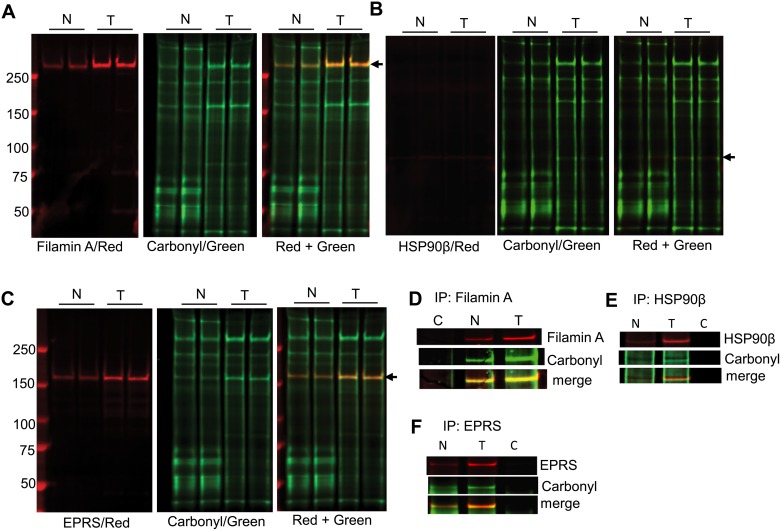
Identification of carbonylated proteins in human breast cancer and adjacent healthy tissue by two-color western. Representative two-color western blots to detect carbonylation in (A) filamin A, (B) HSP90β, and (C) EPRS proteins. Arrow in each panel indicates the overlapping bands between corresponding protein (red) and carbonyl (green). Figure panels (D), (E), and (F) represent the two-color western for immunoprecipitated samples using anti-filamin A, anti-HSP90β and anti-EPRS antibodies respectively in combination with anti-DNP antibody. Carbonylated protein bands were detected using anti-goat 800CW (green) and specific protein bands were detected using donkey anti-rabbit or anti-mouse 680LT (red) as the secondary antibodies. N = adjacent healthy tissue, T = breast tumor tissue.

**Fig 3:**
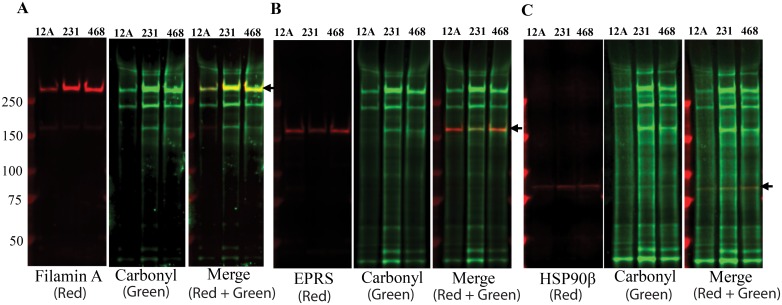
Determination of specific carbonylated proteins in MCF-12A, MDA-MB-231, and MDA-MB-468 cell lines by two-color western. Representative two-color western blots to detect carbonylation in (A) filamin A, (B) EPRS, and (C) HSP90β proteins using anti-filamin A, anti-EPRS and anti-HSP90β antibodies respectively in combination with anti-DNP antibody. Protein carbonyls were detected using anti-goat 800CW (green), and specific protein bands were detected using donkey anti-rabbit or anti-mouse 680LT (red) as the secondary antibodies. 12A = MCF-12A, 231 = MDA-MB-231, 468 = MDA-MB-468.

The bands corresponding to three differentially carbonylated proteins were analyzed by LC-MS/MS (S1 Table). The identity of carbonylated proteins was confirmed by two-color western. Two-color western demonstrated the overlapping carbonyl (green) and protein (red) bands for all three proteins identified by LC-MS/MS analysis (Fig 2A–2C). Based on the proteins identified by LC-MS/MS and overlapping protein and carbonyl bands in a two-color western blot, the three carbonylated proteins were confirmed as 283.47 kDa filamin A, 83.6 kDa heat shock protein 90β (HSP90β) and 170.5 kDa bifunctional glutamate/proline-tRNA ligase (EPRS). We further confirmed the identity of the carbonylated proteins in breast tumor and adjacent normal tissue lysates by immunoprecipitation and two-color western. Two-color western blot analysis of immunoprecipitated proteins showed overlapping protein and carbonyl bands with higher levels of protein carbonylation in tumor tissue compared to adjacent healthy tissue (Fig 2D–2F). We then confirmed the specificity of carbonylation for these proteins by comparing their selective carbonylation in the highly metastatic breast cancer cell line MDA-MB-231, non-metastatic breast cancer cell line MDA-MB-468, and non-cancerous mammary epithelial cell line MCF-12A. Two–color western blot analysis of lysates from these cell lines indicated that filamin A and EPRS had greater levels of carbonylation in MDA-MB-231 and MDA-MB-468 cell lines compared to MCF12A cells (Fig 3A–3C). The level of EPRS and filamin A protein carbonylation in the MDA-MB-468 cell line was slightly lower compared to the MDA-MB-231 cell line. Interestingly, in contrast to the primary human tissue, we did not observe any differences in HSP90β protein carbonylation between the cancerous and non-cancerous cell lines.

### Antioxidant status and autophagy in breast tumor tissue

**Fig 4:**
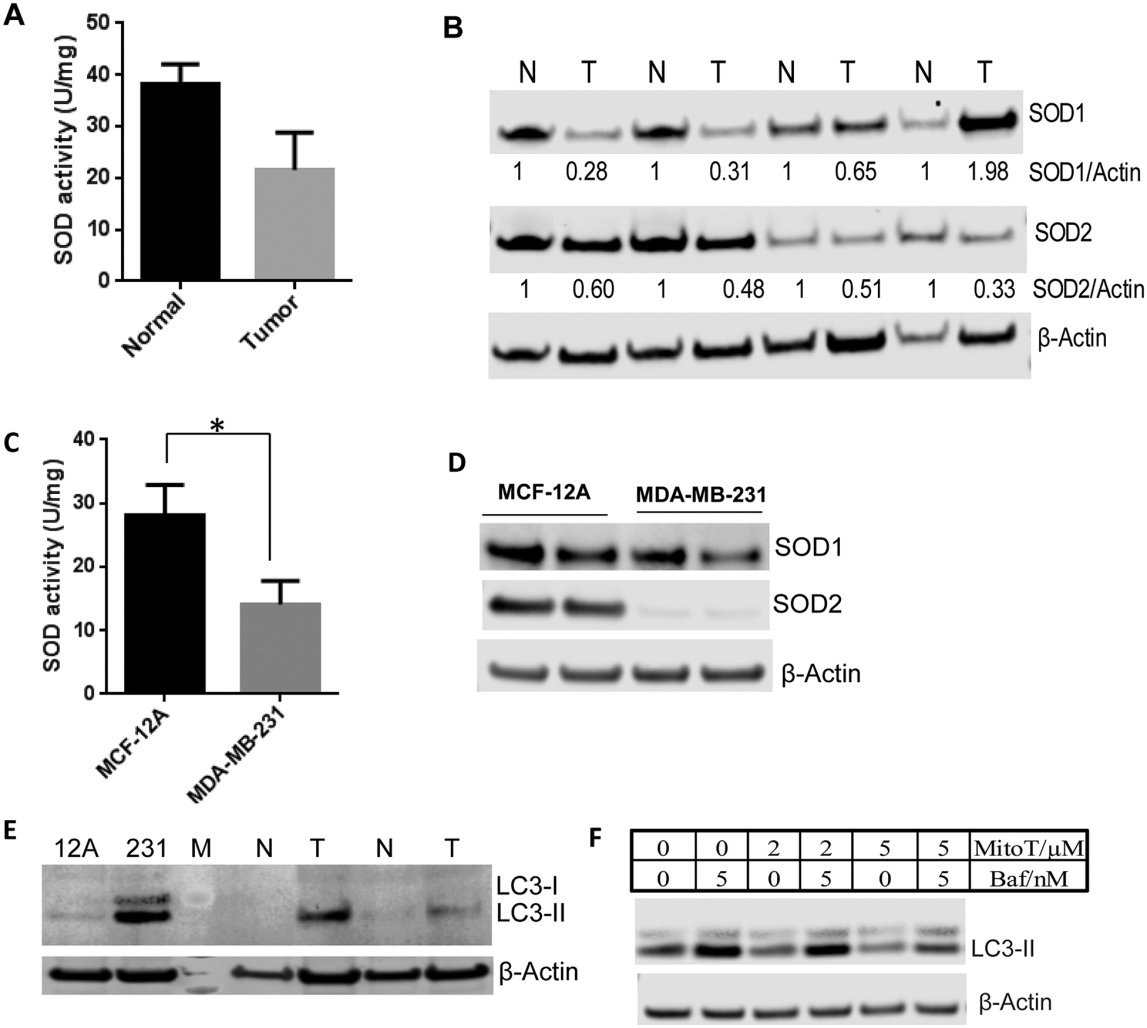
Determination of antioxidant status and autophagy. (A) Quantification of SOD activity in the tumor and adjacent healthy tissue. (B) Representative western blots for SOD1 and SOD2 proteins in breast tumor and adjacent healthy tissue. (C) Quantification of SOD activity in breast cancer MDA-MB-231 and non-cancerous MCF-12A cells. (D) Representative western blots for SOD1 and SOD2 proteins in breast cancer MDA-MB-231 and non-cancerous MCF-12A cell lines. (E) Representative western blot for the basal level of LC3-II in non-cancerous MCF-12A cell line, breast cancer MDA-MB-231 cell line, breast tumor tissue, and adjacent healthy tissue. (F) Representative western blot to demonstrate the effect of antioxidant Mito-Tempol (MitoT) on autophagy using MDA-MB-231 cell lines. β-Actin was used as a loading control in all western blots. M = protein marker, N = adjacent healthy tissue, T = breast tumor tissue, baf = bafilomycin A1, MitoT = Mito-Tempol.

Oxidative stress and antioxidant activity vary among types of cancer and stages of cancer development [3, 18, 19]. To understand if higher levels of carbonylated proteins in breast tumor tissue are associated with lower antioxidant capacity, we measured antioxidant enzyme activity in tumor and adjacent healthy tissue. Superoxide dismutases are the major cellular antioxidant enzymes that scavenge and balance cellular ROS. Previous studies have shown differential SOD activity and oxidative status in early and late stages of human breast cancer tissues, but did not analyze both activity and protein level of SOD enzymes for pairs of tumor and adjacent healthy tissues [5, 20, 21]. We analyzed total SOD activity as well as SOD1 and SOD2 protein levels in tumor and adjacent healthy breast tissue. Lower SOD activity was observed in tumor tissue compared to matched adjacent healthy tissue (Fig 4A). We also observed lower levels of both SOD1 and SOD2 proteins in tumor tissue compared to adjacent healthy tissue (Fig 4B). Of the four tumor tissue samples, only one tumor sample from a 61-year-old subject showed greater SOD1 levels, but SOD2 levels were lower than the respective healthy tissue sample. Despite the greater SOD1 protein level, total protein carbonyl level was still higher in this tumor tissue sample compared to healthy counterpart. Our data indicate that lower SOD activity in tumor tissue and tumor cell lysates compared to their healthy counterparts could be due to reduced SOD protein levels rather than inactivation of SOD proteins.

To extrapolate our findings to cancerous and healthy cell lines, we measured total SOD activity in MDA-MB-231 and MCF-12A cell lines. Like the differences observed in primary tumor and healthy tissues, SOD activity was higher in the non-cancerous MCF-12A cell line compared to MDA-MB-231 breast cancer cell line (Fig 4C). MDA-MB-231 cell lysates also showed lower SOD1 and SOD2 protein levels compared to MCF-12A cells (Fig 4D). Interestingly, compared to MCF-12A cells, MDA-MB-231 cells showed an almost undetectable level of SOD2 by western blot.

Oxidative stress is known to induce autophagy [22]. To understand the basal level of autophagy in tumor tissues and cell lines, we analyzed a common autophagy related protein, LC3-II, by western blot. Both tumor tissues and cell lines showed significantly higher basal levels of LC3-II compared to healthy counterparts (Fig 4E). Our results are consistent with lower antioxidant activity and higher oxidative stress in tumor tissue as indicated by a higher level of carbonylation. To confirm the impact of higher oxidative stress on induction of autophagy flux in MDA-MB-231 cells, we treated MDA-MB-231 cells with the antioxidant Mito-Tempol (MitoT) [23]. Treatment with 5 μM MitoT significantly reduced autophagy flux in MDA-MB-231 cells within 24 hours suggesting that higher autophagy in MDA-MB-231 cells was, at least in part, due to higher oxidative stress (Fig 4F).

## Discussion

Cellular antioxidant systems are critical for maintaining ROS at levels necessary for optimal cellular function. However, cellular antioxidant systems can be altered under certain pathological conditions. Downregulation of several antioxidant genes including *CAT*, *SOD-1*, and *SOD-2* have been reported in cancerous tissue compared to unaffected healthy tissue [24, 25]. Greater levels of total protein carbonylation and reduced levels of antioxidant activity have been reported in breast cancer tissue compared to matched healthy tissue, which can be attributed to the greater levels of ROS and oxidative stress in tumor tissue [21]. Our results provide the first demonstration that total and specific protein carbonylation are greater in breast tumor tissue compared to adjacent healthy tissue. Interestingly, even when total carbonylation was elevated, all proteins were not oxidized to the same extent. Selective carbonylation of three specific proteins was observed in breast tumor tissue and cell lines compared to matched healthy counterparts. We confirmed these specific high molecular weight carbonylated proteins as HSP90β, filamin A, and bifunctional glutamate/proline-tRNA ligase. Earlier studies have shown some selectivity to protein oxidation via carbonylation in cholangiocarcinoma, but mechanisms controlling this selectivity are not well understood [13]. It has been postulated that the level of protein expression, size and higher order structure of protein, number of solvent accessible amino acid residues susceptible to carbonylation, and/or distance of protein/amino acid residues from the ROS generation site may contribute to susceptibility of a specific protein for carbonylation, although more biochemical studies are needed to determine the key drivers [26, 27].

HSP90β is a highly conserved chaperone protein involved in several cellular processes including cell cycle, cell survival, and cellular stress response to maintain cellular homeostasis [28]. HSP90β is also involved in tumor progression and development by stabilizing and protecting mutant oncoproteins from proteasomal degradation [29]. HSP90β is highly expressed in cancerous tissues and high expression of HSP90β was associated with poor prognosis in breast cancer patients [30]. Because of its involvement in stabilizing tumorigenic cells, HSP90β has been a target of cancer therapy. Several posttranslational modifications including phosphorylation, acetylation, nitrosylation, and methylation have been reported to regulate the function of HSP90β but the effect of carbonylation on its function is unknown [31].

Filamin A is a 280 kDa actin-binding scaffold protein that facilitates protein-protein interactions and cellular localization of proteins. Filamin A has been shown to interact with several cellular proteins with diverse functions [32]. Although filamin A is generally known as a cancer promoting protein and its overexpression has been observed in multiple cancers [33], it has also been reported to play a dual role in tumor suppression and progression depending on its subcellular localization. Cytoplasmic localization of full length protein has a tumor promoting effect, while nuclear localization of its 90 kDa C-terminal fragment suppresses tumor growth and invasiveness [34]. Furthermore, elevated levels of filamin A in circulating plasma has been reported to be a specific and sensitive marker for patients with metastatic breast and prostate cancer [35, 36]. It is unknown if filamin A in plasma of breast or prostate cancer patients is carbonylated. In our study, we observed high expression and carbonylation of filamin A in tumor compared to adjacent healthy tissue. The high expression of filamin A in breast tumor tissue may be associated with tumor promoting function. We provide the first evidence for filamin A carbonylation that can be used for follow-on investigations into how its carbonylation, and potentially site-specific carbonylation, impacts its tumor-promoting function.

Bifunctional glutamate/proline-tRNA ligase (EPRS) is an aminoacyl tRNA synthetase of a multisynthetase complex that catalyzes the ligation of glutamate and proline amino acids to their cognate tRNAs. Human EPRS is a 172 kDa protein consisting of two catalytic synthetase cores (ERS and PRS) joined together by a linker. In addition to its function in protein synthesis, it has a non-canonical function serving as a key regulatory gatekeeper of inflammatory gene translation [37, 38]. The specific role of EPRS in cancer is not well understood but its role as a translational silencer of angiogenic factor VEGFA (vascular endothelial growth factor A) indicates that EPRS may influence tumorigenesis [39, 40].

Oxidative stress and protein carbonylation have been detected under several pathological conditions suggesting that carbonylated proteins have a potential role in pathogenesis [41]. Oxidative stress-induced specific protein carbonylation in cancer tissue may cause protein dysfunction and contribute to a poor prognosis. Carbonylation is an irreversible modification to proteins; therefore, identification of total and specifically carbonylated proteins likely indicates a significant imbalance between the levels of free radicals and the mechanisms that would otherwise quench them in healthy cells. Identification of oxidative stress-induced specific protein biomarkers in tumor tissue, identification of specifically carbonylated amino acid residues, and understanding the impact of such protein modifications in the context of cancer initiation and progression may inform future therapies and diagnostics.

## Conclusions

In this study, we investigated selective protein carbonylation in human breast tumor tissue and cell lines compared to their matched healthy counterparts. We identified and confirmed three specific proteins that were carbonylated to a higher extent in breast tumor tissue and cell lines compared to their matched healthy counterparts. While the role of HSP90β in cancer progression is well documented in the literature, the specific roles of filamin A and EPRS in cancer are not well studied. Our recent work on cardiac tissue and purified proteins further confirms that there is some selectivity for oxidative stress-induced carbonylation of proteins and amino acid residues within a protein [16, 27]. We have previously demonstrated that protein functions are affected by the extent of carbonylation. Identification of carbonylated proteins and carbonylation sites within a protein, understanding the importance of such carbonylation in the context of cancer progression, and selectively targeting these proteins to enhance or reduce carbonylation may promote the development of molecular markers of ROS-driven tumorigenesis and targeted therapeutic approaches to mitigate or enhance the selective oxidative damage of such proteins in the tumor. Further studies are required to understand the specificity of *in vivo* protein carbonylation in tumor environments and consequences of such selective carbonylation for protein function.

## Supporting information

S1 Table Protein Identification by LC-MS/MS analysis. (EPS)

## References

[1] Jezierska-Drutel A, Rosenzweig SA, Neumann CA. Role of oxidative stress and the microenvironment in breast cancer development and progression. Adv Cancer Res. 2013;119:107–25. doi: 10.1016/B978-0-12-407190-2.00003-4 23870510 10.1016/B978-0-12-407190-2.00003-4 PMC3950899

[2] Marklund SL, Westman NG, Lundgren E, Roos G. Copper- and zinc-containing superoxide dismutase, manganese-containing superoxide dismutase, catalase, and glutathione peroxidase in normal and neoplastic human cell lines and normal human tissues. Cancer Res. 1982;42(5):1955–61. 7066906

[3] Subapriya R, Kumaraguruparan R, Ramachandran CR, Nagini S. Oxidant-antioxidant status in patients with oral squamous cell carcinomas at different intraoral sites. Clin Biochem. 2002;35(6):489–93. 12413611 10.1016/s0009-9120(02)00340-5

[4] Guner G, Islekel H, Oto O, Hazan E, Acikel U. Evaluation of some antioxidant enzymes in lung carcinoma tissue. Cancer Lett. 1996;103(2):233–9. 8635162 10.1016/0304-3835(96)04226-7

[5] Mahajan M, Tiwari N, Sharma R, Kaur S, Singh N. Oxidative stress and its relationship with adenosine deaminase activity in various stages of breast cancer. Indian J Clin Biochem. 2013;28(1):51–4. doi: 10.1007/s12291-012-0244-5 24381421 10.1007/s12291-012-0244-5 PMC3547448

[6] Visconti R, Grieco D. New insights on oxidative stress in cancer. Curr Opin Drug Discov Devel. 2009;12(2):240–5. 19333869

[7] Storz P. Reactive oxygen species in tumor progression. Front Biosci. 2005;10:1881–96. 15769673 10.2741/1667

[8] Cairns RA, Harris IS, Mak TW. Regulation of cancer cell metabolism. Nat Rev Cancer. 2011;11(2):85–95. doi: 10.1038/nrc2981 21258394 10.1038/nrc2981

[9] Liou GY, Storz P. Reactive oxygen species in cancer. Free Radic Res. 2010;44(5):479–96. doi: 10.3109/10715761003667554 20370557 10.3109/10715761003667554 PMC3880197

[10] Levine RL, Williams JA, Stadtman ER, Shacter E. Carbonyl assays for determination of oxidatively modified proteins. Methods Enzymol. 1994;233:346–57. 8015469 10.1016/s0076-6879(94)33040-9

[11] Uehara H, Rao VA. Metal-mediated protein oxidation: applications of a modified ELISA-based carbonyl detection assay for complex proteins. Pharm Res. 2015;32(2):691–701. doi: 10.1007/s11095-014-1496-y 25182973 10.1007/s11095-014-1496-y

[12] Castegna A, Aksenov M, Thongboonkerd V, Klein JB, Pierce WM, Booze R, et al Proteomic identification of oxidatively modified proteins in Alzheimer’s disease brain. Part II: dihydropyrimidinase-related protein 2, alpha-enolase and heat shock cognate 71. J Neurochem. 2002;82(6):1524–32. 12354300 10.1046/j.1471-4159.2002.01103.x

[13] Thanan R, Oikawa S, Yongvanit P, Hiraku Y, Ma N, Pinlaor S, et al Inflammation-induced protein carbonylation contributes to poor prognosis for cholangiocarcinoma. Free Radic Biol Med. 2012;52(8):1465–72. doi: 10.1016/j.freeradbiomed.2012.01.018 22377619 10.1016/j.freeradbiomed.2012.01.018

[14] Mannello F, Tonti GA, Medda V. Protein oxidation in breast microenvironment: Nipple aspirate fluid collected from breast cancer women contains increased protein carbonyl concentration. Cell Oncol. 2009;31(5):383–92. doi: 10.3233/CLO-2009-0483 19759418 10.3233/CLO-2009-0483 PMC4619038

[15] Nathan FM, Singh VA, Dhanoa A, Palanisamy UD. Oxidative stress and antioxidant status in primary bone and soft tissue sarcoma. BMC Cancer. 2011;11:382 doi: 10.1186/1471-2407-11-382 21871117 10.1186/1471-2407-11-382 PMC3178545

[16] Aryal B, Jeong J, Rao VA. Doxorubicin-induced carbonylation and degradation of cardiac myosin binding protein C promote cardiotoxicity. Proc Natl Acad Sci U S A. 2014;111(5):2011–6. doi: 10.1073/pnas.1321783111 24449919 10.1073/pnas.1321783111 PMC3918758

[17] Mehrabi S, Partridge EE, Seffens W. Oxidatively modified proteins in the serous subtype of ovarian carcinoma. Biomed Res Int. 2014;2014:585083 doi: 10.1155/2014/585083 24795885 10.1155/2014/585083 PMC3985143

[18] Gopcevic KR, Rovcanin BR, Tatic SB, Krivokapic ZV, Gajic MM, Dragutinovic VV. Activity of superoxide dismutase, catalase, glutathione peroxidase, and glutathione reductase in different stages of colorectal carcinoma. Dig Dis Sci. 2013;58(9):2646–52. doi: 10.1007/s10620-013-2681-2 23625289 10.1007/s10620-013-2681-2

[19] Kocot J, Kielczykowska M, Dabrowski W, Pilat J, Rudzki S, Musik I. Total antioxidant status value and superoxide dismutase activity in human colorectal cancer tissue depending on the stage of the disease: a pilot study. Adv Clin Exp Med. 2013;22(3):431–7. 23828685

[20] Hasan HR, Mathkor TH, Al-Habal MH. Superoxide dismutase isoenzyme activities in plasma and tissues of Iraqi patients with breast cancer. Asian Pac J Cancer Prev. 2012;13(6):2571–6. 22938422 10.7314/apjcp.2012.13.6.2571

[21] Panis C, Victorino VJ, Herrera AC, Freitas LF, De Rossi T, Campos FC, et al Differential oxidative status and immune characterization of the early and advanced stages of human breast cancer. Breast Cancer Res Treat. 2012;133(3):881–8. doi: 10.1007/s10549-011-1851-1 22048816 10.1007/s10549-011-1851-1

[22] Malaviya R, Laskin JD, Laskin DL. Oxidative stress-induced autophagy: role in pulmonary toxicity. Toxicol Appl Pharmacol. 2014;275(2):145–51. doi: 10.1016/j.taap.2013.12.022 24398106 10.1016/j.taap.2013.12.022 PMC4455034

[23] Trnka J, Blaikie FH, Logan A, Smith RA, Murphy MP. Antioxidant properties of MitoTEMPOL and its hydroxylamine. Free Radic Res. 2009;43(1):4–12. doi: 10.1080/10715760802582183 19058062 10.1080/10715760802582183 PMC2645131

[24] Wu Z, Boonmars T, Boonjaraspinyo S, Nagano I, Pinlaor S, Puapairoj A, et al Candidate genes involving in tumorigenesis of cholangiocarcinoma induced by Opisthorchis viverrini infection. Parasitol Res. 2011;109(3):657–73. doi: 10.1007/s00436-011-2298-3 21380578 10.1007/s00436-011-2298-3

[25] De Palma G, Mozzoni P, Acampa O, Internullo E, Carbognani P, Rusca M, et al Expression levels of some antioxidant and epidermal growth factor receptor genes in patients with early-stage non-small cell lung cancer. J Nucleic Acids. 2010;2010. 10.4061/2010/147528 PMC2911612 20700416

[26] Maisonneuve E, Ducret A, Khoueiry P, Lignon S, Longhi S, Talla E, et al Rules governing selective protein carbonylation. PLoS One. 2009;4(10):e7269 doi: 10.1371/journal.pone.0007269 19802390 10.1371/journal.pone.0007269 PMC2751825

[27] Kryndushkin D, Wu WW, Venna R, Norcross MA, Shen RF, Rao VA. Complex Nature of Protein Carbonylation Specificity After Metal-Catalyzed Oxidation. Pharm Res. 2017;34(4):765–79. doi: 10.1007/s11095-017-2103-9 28150167 10.1007/s11095-017-2103-9

[28] Young JC, Moarefi I, Hartl FU. Hsp90: a specialized but essential protein-folding tool. J Cell Biol. 2001;154(2):267–73. doi: 10.1083/jcb.200104079 11470816 10.1083/jcb.200104079 PMC2150759

[29] Grbovic OM, Basso AD, Sawai A, Ye Q, Friedlander P, Solit D, et al V600E B-Raf requires the Hsp90 chaperone for stability and is degraded in response to Hsp90 inhibitors. Proc Natl Acad Sci U S A. 2006;103(1):57–62. doi: 10.1073/pnas.0609973103 16371460 10.1073/pnas.0609973103 PMC1325013

[30] Pick E, Kluger Y, Giltnane JM, Moeder C, Camp RL, Rimm DL, et al High HSP90 expression is associated with decreased survival in breast cancer. Cancer Res. 2007;67(7):2932–7. doi: 10.1158/0008-5472.CAN-06-4511 17409397 10.1158/0008-5472.CAN-06-4511

[31] Li J, Soroka J, Buchner J. The Hsp90 chaperone machinery: conformational dynamics and regulation by co-chaperones. Biochim Biophys Acta. 2012;1823(3):624–35. doi: 10.1016/j.bbamcr.2011.09.003 21951723 10.1016/j.bbamcr.2011.09.003

[32] Nakamura F, Stossel TP, Hartwig JH. The filamins: organizers of cell structure and function. Cell Adh Migr. 2011;5(2):160–9. doi: 10.4161/cam.5.2.14401 21169733 10.4161/cam.5.2.14401 PMC3084982

[33] Yue J, Huhn S, Shen Z. Complex roles of filamin-A mediated cytoskeleton network in cancer progression. Cell Biosci. 2013;3(1):7 doi: 10.1186/2045-3701-3-7 23388158 10.1186/2045-3701-3-7 PMC3573937

[34] Savoy RM, Ghosh PM. The dual role of filamin A in cancer: can’t live with (too much of) it, can’t live without it. Endocr Relat Cancer. 2013;20(6):R341–56. doi: 10.1530/ERC-13-0364 24108109 10.1530/ERC-13-0364 PMC4376317

[35] Alper O, Stetler-Stevenson WG, Harris LN, Leitner WW, Ozdemirli M, Hartmann D, et al Novel anti-filamin-A antibody detects a secreted variant of filamin-A in plasma from patients with breast carcinoma and high-grade astrocytoma. Cancer Sci. 2009;100(9):1748–56. doi: 10.1111/j.1349-7006.2009.01244.x 19594548 10.1111/j.1349-7006.2009.01244.x PMC2788299

[36] Narain NR, Diers AR, Lee A, Lao S, Chan JY, Schofield S, et al Identification of Filamin-A and -B as potential biomarkers for prostate cancer. Future Sci OA. 2017;3(1):Fso161. 10.4155/fsoa-2016-0065 PMC5351499 28344825

[37] Kim S, You S, Hwang D. Aminoacyl-tRNA synthetases and tumorigenesis: more than housekeeping. Nat Rev Cancer. 2011;11(10):708–18. doi: 10.1038/nrc3124 21941282 10.1038/nrc3124

[38] Sampath P, Mazumder B, Seshadri V, Gerber CA, Chavatte L, Kinter M, et al Noncanonical function of glutamyl-prolyl-tRNA synthetase: gene-specific silencing of translation. Cell. 2004;119(2):195–208. doi: 10.1016/j.cell.2004.09.030 15479637 10.1016/j.cell.2004.09.030

[39] Park SG, Schimmel P, Kim S. Aminoacyl tRNA synthetases and their connections to disease. Proc Natl Acad Sci U S A. 2008;105(32):11043–9. doi: 10.1073/pnas.0802862105 18682559 10.1073/pnas.0802862105 PMC2516211

[40] Ray PS, Fox PL. A post-transcriptional pathway represses monocyte VEGF-A expression and angiogenic activity. Embo j. 2007;26(14):3360–72. doi: 10.1038/sj.emboj.7601774 17611605 10.1038/sj.emboj.7601774 PMC1933405

[41] Levine RL. Carbonyl modified proteins in cellular regulation, aging, and disease. Free Radic Biol Med. 2002;32(9):790–6. 11978480 10.1016/s0891-5849(02)00765-7

